# Observations on an Universal Anchylosis

**Published:** 1802-11-01

**Authors:** 

**Affiliations:** Professor of the School of Medicine, at Paris


### OBSERVATIONS *on an universal* ANCHYLOSIS; *by* Cit. PERCY, *Professor of the School of Medicine, at Paris.*

THE Author having ſtated and compared all the Caſes analagous to that of which he gives an account, remarks, That no caſe of ſuch a complete Anchyloſis has ever been recorded in the Annals of Medicine, and that no Collection ſeems to poſſeſs ſo univerſal a concretion of the bones as that which is at preſent depoſited in the Cabinet of the Medical School at Paris. The ſkeleton, in fact, conſiſts of only one piece, though by the removal and the anatomical preparation of it, ſeveral bones have been ſeparated; as, for inſtance, the vertebræ lumborum and ſacrales, the os femoris, and humeri of the right ſide; but the traces of their having been preternaturally united may ſtill be ſeen. The back bone is but little bent, the pelvis however is conſiderably prominent; the thigh bone forms an acute angle with the tibia and fibula; the right hand is turned inward, and the left hand outward; all the fingers are curvated; the bones themſelves are very light, and their calcareous matter is very brittle. The hiſtory of the diſeaſe, by which this Anchyloſis was produced, is as follows.

Francis Maurice Mercier Simorre was born in the year 1752; when he attained the fifteenth year of his age he entered into the military profeſſion, ſerved twenty-one years, and was promoted to the rank of a Captain of Infantry. During this time he made three campaigns in the Corſican war. Having been a long time encamped on marſhy grounds, and in a moiſt and cold atmoſphere, he began to perceive an acute pain in the great toes and in the ankles, where the ſkin was red and inflamed. This affection being taken for the gout, and treated accordingly, the pain ceaſed, but at the end of ſome months, an inflammation of the eyes ſupervened, which was likewiſe removed by proper remedies. During ſeveral years however, the gouty complaint, and that ophthalmy, affected the patient alternately in the beginning of ſpring. Theſe two diſeaſes, which returned periodically, having laſted much longer during two ſucceeding years, the ſight of the patient became extremely weak, and he could not walk without the aſſiſtance of a guide. In the year 1786 all his joints were at once attacked; the feet, the knees, and all parts of the inferior extremities began to become anchylotic. Mr. Simorre quitted the ſervice about this time, and retired to Metz, where he ſubſiſted upon a ſmall penſion.

The diſeaſe increaſed daily, the arms, the head, the vertebra, and even the maxilla inferior were cemented together. The unhappy patient was nothing, according to his own expreſſion, but a living carcaſe. His ſenſibility was at the ſame time very great, as he ſuffered the moſt excruciating pains. Four years he paſſed in an arm chair, without ever ſhutting his eyes; he was afterwards conveyed with much difficulty into a bed, where he remained two years longer, without being able to obtain even a moment's ſleep. His poſture was ſitting, to which circumſtance the ſituation of the bones in the ſkeleton is to be aſcribed. He had entirely loſt his ſight, the cornea and the lens cryſtallina being opaque. In the year 1792, the joints, which had hitherto been tumid, began to grow leſs, and the pains were a little aſſuaged; but there ſupervened regularly twice a month an eryſipelatous inflammation, attended with fever, and an itching, which proved the more intolerable, as the patient could not relieve it by ſcratching. This complaint reſiſted all poſſible remedies. When the anchyloſis was entirely eſtabliſhed in all the joints, the patient could be better managed than before. He had all his teeth remaining, but was obliged to ſuck in his fluid nouriſhment. The inciſive teeth being afterwards drawn out, he could receive more ſolid food, make himſelf underſtood, and throw out his ſpittle. His figure was very expreſſive and ſingular, his hair black, and noſe aqualine. The muſcles of his face were in continual motion; his body was extremely emaciated; during reſpiration, his ſides and ſternum did not move; the inſpiration was attended with much noiſe, and the pulſe beat 60 or 63 times in a minute; he had no ſenſible perſpiration; his ſtools were numerous, and his diureſis abundant; the urine ſhewed no difference from that of a healthy man. When he died he was about 50 years old. On opening the body nothing remarkable was diſcovered; the left lungs adhered to the pleura, and had ſome ſuppurating tubercles.

